# Optimization of Whole-Genome Resequencing Depth for High-Throughput SNP Genotyping in *Litopenaeus vannamei*

**DOI:** 10.3390/ijms252212083

**Published:** 2024-11-11

**Authors:** Pengfei Lin, Yang Yu, Zhenning Bao, Fuhua Li

**Affiliations:** 1 Key Laboratory of Breeding Biotechnology and Sustainable Aquaculture (CAS), Institute of Oceanology, Chinese Academy of Sciences, Qingdao 266071, China; lpf1870388182@163.com (P.L.); 17864277709@163.com (Z.B.); fhli@qdio.ac.cn (F.L.); 2 Laboratory for Marine Biology and Biotechnology, Qingdao Marine Science and Technology Center, Qingdao 266071, China; 3 University of Chinese Academy of Sciences, Beijing 100049, China

**Keywords:** *Litopenaeus vannamei*, sequencing depth, SNP quantity and quality, whole-genome resequencing

## Abstract

The application of whole-genome resequencing in genetic research is rapidly expanding, yet the impact of sequencing depth on data quality and variant detection remains unclear, particularly in aquaculture species. This study re-sequenced 31 *Litopenaeus vannamei* (*L. vannamei*) samples at over 28× sequencing depth using the Illumina NovaSeq system and down-sampled the data to simulate depths from 0.5× to 20×. Results showed that when the sequencing depth was below 10×, the number of SNP identifications increased sharply with the rise in depth, with single nucleotide polymorphisms (SNPs) detected at 10× accounting for approximately 69.16% of those detected at 20×. The genotyping accuracy followed a similar trend to SNP detection results, being approximately 0.90 at 6×. Further analyses showed that the main cause of genotyping errors was the misidentification of heterozygous variants as homozygous variants. Therefore, considering both the quantity and quality of SNPs, a sequencing depth of 10× is recommended for whole-genome studies and genetic mapping, while a depth of 6× is more cost-effective for population structure analysis. This study underscores the importance of selecting optimal sequencing depth to ensure reliable variant detection and high data quality, providing valuable guidance for whole-genome resequencing in shrimp and other aquatic species.

## 1. Introduction

Whole-genome resequencing is the method of sequencing the genomes of different individuals based on a known reference genome and analyzing the differences between individuals or populations [1]. It offers utility in the mapping of quantitative trait loci (QTLs), genome-wide association studies (GWAS) of economic traits, and the analysis of population evolution. A large number of single nucleotide polymorphisms (SNPs), structural variations (SVs), insertion-deletions (InDels), and copy number variations (CNVs) can be identified by comparing individual sequences to the reference sequence. The advent of high-throughput sequencing technology and the concomitant development of bioinformatics tools have made resequencing an effective method for genetic research in reference-available species. High-quality sequencing data not only yields a wealth of information but also enhances the precision of gene identification, thereby facilitating more sophisticated genomic analysis [2]. However, the financial burden of sequencing frequently constrains the number of individuals that can be sequenced, which, in turn, impacts the experimental design and outcomes of biological research. Despite the considerable reduction in sequencing costs resulting from advances in next- and third-generation sequencing technology, cost remains an important factor, especially for aquaculture species with large numbers of offspring. The sequencing depth represents a major challenge in terms of sequencing cost. It can be defined as the ratio of the total sequencing bases to the genome size or the average number of times each base in the genome is sequenced [3]. Furthermore, sequencing depth is positively correlated with the ability to detect rare mutations. In order to achieve an appropriate balance between sequencing cost and data quality, it is necessary to conduct a detailed evaluation of the effect of sequencing depth on the quality and quantity of variant discovery. This will enable the determination of the optimal sequencing depth for genetic studies.

The depth of sequencing in genome resequencing analysis can impact the number of variants identified, the precision of genotyping results, and the cost of the project. The appropriate selection of sequencing depth can improve the accuracy of both mutation detection and mutation frequency estimation. This facilitates the construction of an accurate genomic variant map and enhances the detection of genetic variation. For example, a report on pigs indicates that 10× is the ideal practical depth for achieving platform coverage and accurately detecting variants, while 4× is the minimum required for reasonable sequencing quality [4]. Moreover, an average sequencing depth of approximately 11× to 12× in Tibetan sheep is sufficient for the identification of a substantial number of SNPs, thereby providing a robust dataset for the analysis of genetic diversity and selective traits [5]. These data can facilitate subsequent research aimed at identifying selective regions [6,7] and can also serve as a reasonable point of reference with regard to sequencing depth in other species. This, in turn, has the potential to yield significant economic benefits for numerous animal breeding studies [8,9,10].

However, it is worth noting that most studies examining the influence of sequencing depth on genomic data analysis have predominantly focused on human samples and model organisms. Similar studies on non-model species are limited in scope. There has been a notable paucity of research conducted specifically on aquatic species, which presents a significant challenge when dealing with the large size of the shrimp genome. The Pacific white shrimp (*Litopenaeus vannamei*) is a species that is cultivated worldwide. A reference genome has been previously published [11]. Despite the fact that several whole-genome resequencing analyses have been conducted [12,13], there is currently no systematic analysis of the optimal sequencing depth. The lack of research in the field of aquaculture underscores the necessity of ascertaining the optimal sequencing depth for shrimp. By addressing this gap, it would be possible to obtain more precise and comprehensive data for genetic variation and functional analysis. In addition, the insights gained from these studies can be applied to other aquatic species, especially invertebrates. These findings provide a valuable reference and guidance for studies on genome resequencing in aquaculture.

In this study, the whole genome of 31 representative individuals of *Litopenaeus vannamei* was sequenced, all of which were sequenced to a depth of more than 28×. To further address the trade-off between SNP quality and cost, we investigated the association between sequencing depth and biological outcomes in terms of variant discovery capacity and variant quality. In addition, we investigated the effect of heterozygosity on genotyping accuracy. These findings will provide valuable guidance for the design of whole-genome resequencing studies and accelerate genetic research in *L. vannamei* and other aquatic species.

## 2. Results

### 2.1. Summary of the Whole-Genome Resequencing Data

**Figure 1:**
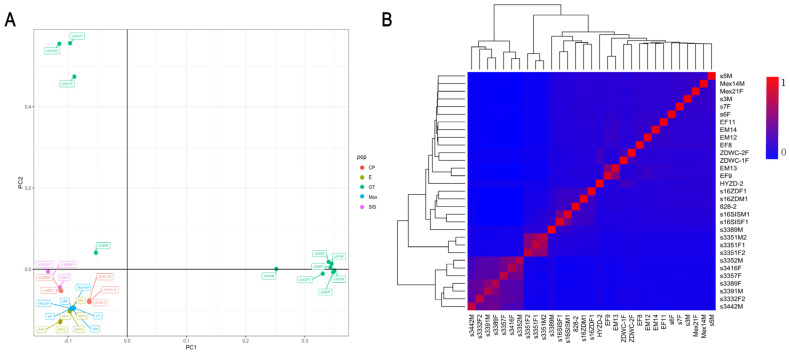
Population structure of sequenced individuals. (**A**) Principal component analysis (PCA) of individuals. Different colors indicate different species of shrimp. PC1: first principal component; PC2: second principal component. (**B**) Heatmap of all 31 sequenced individuals using the molecular relationship matrix. The exact genomic relationship between two individuals is shown in each small lattice. The larger the value within 0–1, the closer the kinship.

The resequencing data from 31 samples are summarized in Table 1. Each individual reached a depth of more than 28×, with coverage exceeding 90.00% of the whole genome. Q20 scores were generally greater than 90.00%, while Q30 scores were generally more than 85.00%, and GC content ranged from 40.00% to 50.00%. Figure 1A illustrats a clear separation between the different breeds. The heatmap of genomic relationships between individuals, as shown in Figure 1B, indicates that individuals from each taxon are largely clustered together.

### 2.2. Discovery of Variants Based on Different Depth

**Figure 2:**
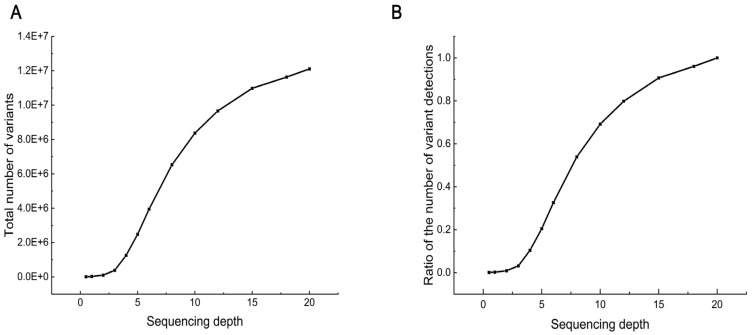
SNP discovery capacity at different sequencing depths. (**A**) Total number of variants (SNPs only) discovered in different sequencing depths. (**B**) Ability to detect variants. The number of variants found at each depth as a proportion of the total number of variants found at the maximum depth.

After random down-sampling, the number of SNPs found at each depth showed an approximately S-shaped increase with increasing sequencing depth (Figure 2A), and the variant discovery power at different depths showed the same trend as the number of SNPs. Specifically, when the sequencing depth was less than 3×, the number of SNPs found increased slowly and gradually from almost 8000 to 380,000. Subsequently, the number of SNPs found increased rapidly as the depth increased from 3× to 10×, with the increase slowing after 10×. At 10×, about 8.37 million SNPs were detected, accounting for 69.16% of the variation at 20× sequencing depths (Figure 2B). When the depth reached 15×, the variant detection rate exceeded 90.00%.

### 2.3. SNP Genotyping Accuracy at Different Depths

**Figure 3:**
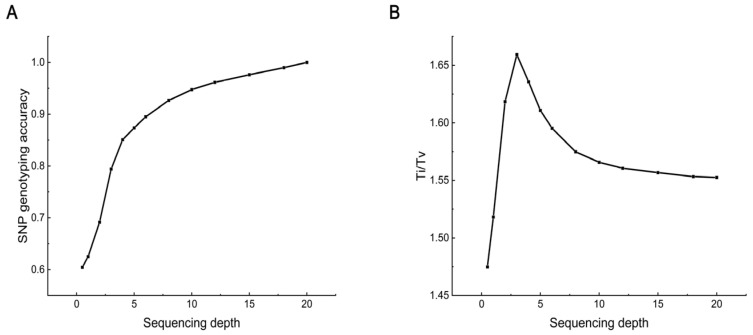
SNP genotyping accuracy at different sequencing depths. (**A**) SNP genotyping accuracy. The proportion of SNPs with identical genotyping when the genotyping results at the corresponding sequencing depth were compared to the genotyping results of the 20× data. (**B**) Ti/Tv ratio. The proportion of variants observed as transitions (between purines or between pyrimidines) to transversions (between purines and pyrimidines).

Using the 20× data results as a reference, the accuracy of SNP genotyping at different sequencing depths is shown in Figure 3A. Not surprisingly, accuracy increased with depth. As the depth increased from 0.5× to 18×, accuracy increased from 0.60 to 0.99. From 1× to 4×, accuracy increased rapidly with sequencing depth, showing a clear upward trend. However, there was a tipping point at 4×, where the effect of further increases in depth began to diminish and gradually level off. At 6×, accuracy was approximately 0.90.

The transitions/transversions (Ti/Tv) ratio was another parameter used to measure the accuracy of genotyping. As shown in Figure 3B, the Ti/Tv ratio did not change significantly. At low depths, the ratio fluctuated greatly. With increased sequencing depth, the ratio gradually tended to stabilize and remained generally unchanged at about 1.55.

### 2.4. Genotyping Errors and Proportions of Heterozygotes and Homozygotes

**Figure 4:**
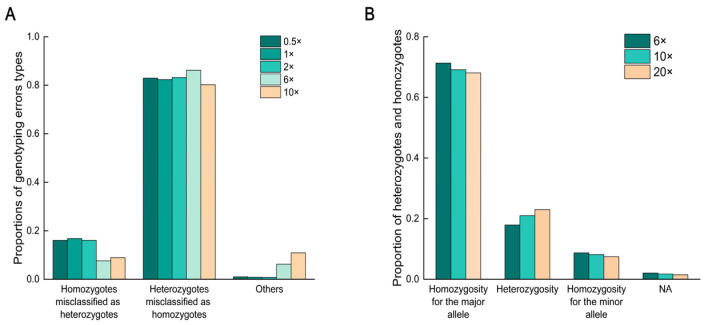
Proportions of genotyping error types and the proportion of heterozygotes and homozygotes at different sequencing depths. (**A**) Proportions of different error types at five sequencing depths. (**B**) Proportion of heterozygotes and homozygotes at three sequencing depths. NA indicates missing or unavailable data.

Due to the low accuracy at low sequencing depths, we further analyzed the causes of genotyping errors at five different sequencing depths (0.5×, 1×, 2×, 6×, and 10×). The results indicated that most genotyping errors were due to misclassifying heterozygotes as homozygotes (Figure 4A), accounting for over 80.00% of all error types. We also calculated the proportions of heterozygotes and homozygotes at three sequencing depths (6×, 10×, 20×). Genotyping results were similar at all three sequencing depths, with heterozygotes accounting for approximately 20.00% of the genome-wide genotyping data (Figure 4B). This finding suggests that higher genomic heterozygosity might have contributed to higher genotyping error rates, and that other species with high heterozygosity might face similar challenges at low sequencing depths.

### 2.5. Evaluation of SNP Detection by Annotation

**Figure 5:**
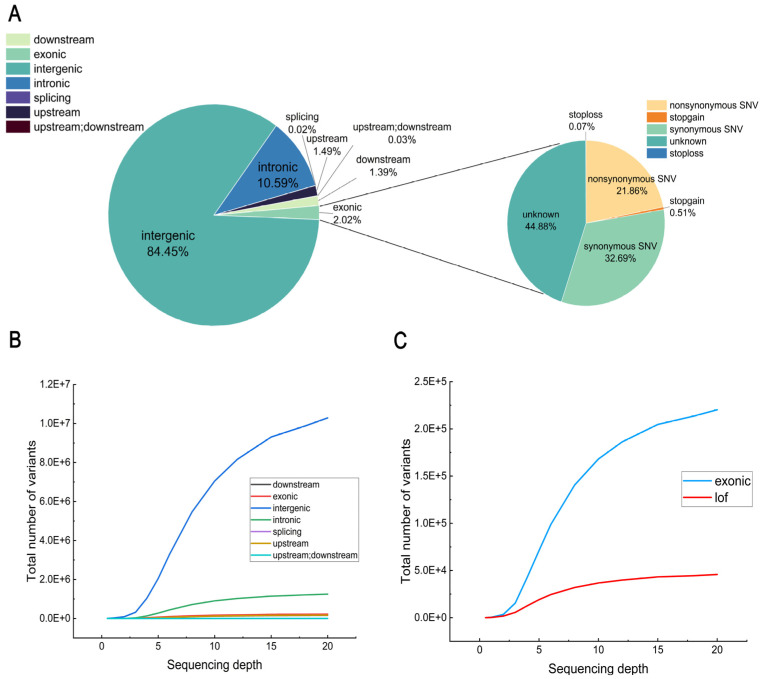
Evaluation of SNP detection by annotation. (**A**) Proportion of different variant types, with large circles indicating the proportion of SNPs in each region of the genome and small circles indicating the proportion of each variant type in exons. (**B**) Trends in the number of types in which the variation resided with depth, such as exons. (**C**) Changes with depth in the number of SNPs in exonic regions and in the number of variants identified as potential loss-of-function (LoF) variants that met specific criteria.

All variants were annotated using ANNOVA (https://annovar.openbioinformatics.org/en/latest/, accessed on 6 March 2024). The results showed that a total of seven variant types were identified in the annotated gene regions, with the highest number of intergenic variants accounting for approximately 84.45%. Intronic variants accounted for 10.59%, and exonic variants accounted for only 2.02% (Figure 5A). We further characterized the types of variants identified as loss-of-function (Lof) in exons, of which non-synonymous single nucleotide variants (SNVs) accounted for a large proportion. The stopgain and stoploss types accounted for only a small fraction (Figure 5A). The number of variants in different regions of the genome increased with sequencing depth (Figure 5B), as did the number of exon variants and Lof findings (Figure 5C). Similar to the total number of SNPs, the number of different types of variants increased rapidly in the early stages and stabilized in the later stages.

## 3. Discussion

As the cost of sequencing decreases, the application of whole-genome resequencing becomes increasingly common. Genomic selection (GS) and genome-wide association studies (GWAS) are also gradually adopting whole-genome resequencing for their studies [14,15]. In order to ensure the validity of data while reducing costs, exploring the optimal sequencing depth has become particularly important. In aquaculture species, whole-genome resequencing has been applied to several species, such as large yellow croaker (*Larimichthys crocea*) [16], bay scallop (*Argopecten irradians*) [17], sea cucumber (*Apostichopus japonicus*) [18], cultivated gilthead seabream (*Sparus aurata*) [19,20], Pacific oyster (*Crassostrea gigas*) [21,22], Atlantic salmon (*Salmo salar*) [23], and others. In this study, we focused on whole-genome resequencing technology in shrimp, aiming to explore the relationship between sequencing depth, variant detection, and variant quality. Based on our findings, researchers can more effectively choose the appropriate sequencing depth to improve data accuracy and quality, which will be useful for shrimp genomics research and aquaculture applications.

The accuracy and number of variants identified are contingent upon the sequencing depth. In previous simulations and human datasets, researchers have demonstrated that sequencing depths of 5–10× are sufficient to detect frequent mutations [24]. Our results showed that the number of variants increased as the sequencing depth increased. The number of identified SNPs increased rapidly from 3× to 10×, and the identification rate reached 90.66% at 15×. Consequently, 10×~15× was needed to obtain comprehensive variants in shrimp. Although high-depth sequencing can yield more information, recent studies have demonstrated that employing lower-depth sequencing is a more efficient and powerful approach for large populations. In scenarios involving large sample sizes, conducting low-depth sequencing on a substantial number of individuals proves to be a more efficient approach than high-depth sequencing, particularly for the detection of rare mutations. Moreover, the cost required for high-depth sequencing methods is significantly higher than that for low-depth sequencing methods [25]. It thus follows that the Thousand Human Genome Project employed 4× data in an effort to identify variants associated with a spectrum of complex human diseases [26]. For the purpose of evaluating genome-wide genetic variation, a depth of 10× or higher has been used in species such as sheep and catfish [27,28]. Similar depths of 10× are used to detect selection characteristics in pig populations [29,30]. However, due to the high complexity and large number of repetitive sequences that typically characterize aquatic invertebrate genomes, it remains uncertain whether reliable data can be obtained using the same sequencing depth. The choice of analysis method determines the optimal sequencing depth, which is essential for ensuring data accuracy and the validity of the study.

An essential factor in determining the quality of SNPs is the Ti/Tv ratio [31], which is predicted to be between 2.1 and 2.2 for genome-wide variation. When detected variants show a rate close to the expected rate of random substitutions (e.g., ~0.5), it suggests low-quality variant calling [32]. Meanwhile, elevated Ti/Tv ratios are typically indicative of enhanced precision in SNP calling [33]. The results obtained for the Ti/Tv ratio were not in accordance with the anticipated ratio. The Ti/Tv ratio stabilized at around 1.55 as the sequencing depth increased, stabilizing at a sequencing depth of 10×, which resulted in a lower value than that in previous reports. A previous study on shrimp shows that the transitions/transversions ratio in the biallelic SNP set is found to be 1.63 [12]. This may be attributed to the influence of biological factors on the variation in Ti/Tv ratios across species, with these ratios exhibiting notable differences depending on genomic regions and functions. For instance, in the context of human exome sequencing data, Ti/Tv ratios are typically observed to be approximately 3.0, while outside of exome regions, they are seen to be around 2.0 [34]. Additionally, Ti/Tv ratios have been shown to vary between synonymous and non-synonymous SNPs [35]. Accordingly, it is essential to take into account species-specific differences and the classification of SNPs according to their genomic locations and functions when calculating Ti/Tv ratios.

It is evident that the accuracy of genotyping plays a pivotal role in determining the outcome of the experiment. However, this crucial aspect was previously overlooked. We found that the accuracy at depths below 6× was too low for further genetic analysis. A depth of 10× was required to obtain highly accurate results. The genotyping error rate of Next-Generation Sequencing (NGS) data increases with decreasing genome coverage, and coverage is positively correlated with sequencing depth [36,37]. Previous studies have validated the genotyping accuracy of NGS using the genotyping chip PorcineSNP60 array. These results showed that the majority of genotyping errors can be attributed to low sequencing coverage [32].

By calculating the proportion of heterozygotes and homozygotes in the data at different depths, we found that the proportion of heterozygotes was about 20.00%. Based on the accuracy data, we calculated the proportion of error types at the five sequencing depths and found that heterozygotes were misclassified as homozygotes in a large proportion. Similar results for humans also indicate that heterozygotes being misclassified as homozygotes is the most common error type [38]. A distinguishing feature of the genomes of some aquaculture species is the relatively high level of heterozygosity [11,39]. A report on the highly heterozygous Pacific oyster assessed the impact of coverage on genotype detection and accuracy. The findings indicated that 15× was an appropriate level of coverage for obtaining a sufficient number of high-precision SNPs [40]. Overall, this phenomenon is not exclusive to shrimp. Similarly, other aquatic species with relatively high genomic heterozygosity may face similar challenges at low sequencing depths. Low sequencing depth severely affects the accuracy of genotyping in these species, with the main consequence being the misclassification of heterozygotes as homozygotes. For example, many aquatic species such as crabs [41], oysters [39], and sea urchins [42] have relatively high levels of heterozygosity, leading to genotyping error patterns similar to those observed in shrimp at low sequencing depths. To improve genotyping accuracy, particularly when working with species that exhibit a high degree of heterozygosity, it is imperative to employ higher sequencing depths. This not only reduces the occurrence of genotyping errors but also provides more reliable genetic data, thereby facilitating deeper genetic research and practical applications.

Although high depth is needed for individual genotyping, low-depth genome resequencing in a large population has also been demonstrated to be an accurate and efficient method for genotyping. The fundamental process underlying low-depth sequencing is genotype imputation, which utilizes reference panels, linkage disequilibrium patterns, and statistical algorithms to accurately infer genotypes from limited data. For example, statistical methods were used to infer the missing genotypes from the known haplotype reference data or the shared haplotype information among a large number of samples. Sequencing to Imputation through Constructing Haplotypes (STITCH, version 1.0.0) software, developed by Davies, uses hidden Markov models and the EM algorithm to estimate ancestral haplotypes in study populations [43]. It utilizes these ancestral haplotypes to populate missing genotypes in low-depth resequencing data, thereby partially addressing the dearth of high-quality reference haplotype datasets in non-human genome studies. In mice and pigs, it has been demonstrated that low-depth sequencing data can be effectively used for genotyping and genome-wide association analysis in populations exceeding 1000 individuals [44,45]. In addition, in important livestock species such as pigs and chickens, various research results based on low-depth data analysis have been reported [46,47,48], providing novel methodologies and concepts for the genotyping of large samples of low depth in livestock species. Nevertheless, the assessment of heterozygosity at low depth continues to present a significant challenge. A study on the relationship between sequencing depth and variant detection showed that homozygous variants reach saturation at a sequencing depth of 15×, while heterozygous variants require a depth of 30× to reach saturation [49]. It can be observed that the accurate detection of heterozygotes necessitates a higher depth of sequencing. While techniques such as quality filtering and the utilization of diverse algorithms can be employed to enhance the precision of low-depth sequencing data, the results may vary for different species. Thus, accurate determination of genotypes at low sequencing depths remains an unsolved challenge for species with relatively high genomic heterozygosity.

## 4. Methods

### 4.1. Whole-Genome Resequencing

The *L. vannamei* samples used in this study were composed of 6 wild-captured individuals, and 25 individuals came from 4 commercial lines. These individuals belong to five groups: the Charoen Pokphand group (CP) from the commercial broodstock of Charoen Pokphand Foods Company in Thailand; the wild Mexico group (Mex) from the coast of Baja California Sur, Mexico; the Ecuador group (E) from the commercial broodstock in Ecuador; the SIS group (SIS) from the commercial broodstock of Shrimp Improvement System Company in the United States; and the Guangtai No. 1 group (GT) from the commercial broodstock in China. The DNA was extracted using a Tiangen genomic DNA kit. The NGS libraries were constructed for each individual, and then the libraries were sequenced on the NovaSeq system (Illumina, San Diego, CA, USA)with a paired-end sequencing platform. All raw sequencing data were first trimmed using SOAPnuke (version 2.1.7) and indexed using BWA (version 0.7.17) [50], and high-quality reads were mapped to the reference genome sequence using BWA-MEM with default parameters. SAMtools (version 1.18) [51] was used to import BAM (Binary Alignment/Map) files for read sequencing, format conversion, and reference index building.

### 4.2. Construction of Samples with Different Sequencing Depths

To create samples with different sequencing depths, the BAM files were randomly down-sampled using the Picard DownsampleSam (version 2.27.3) tool. The down-sampling was performed based on different ratios, taking into account the read length and sequencing depth of the original mapped BAM files. The gradient depths selected for further analysis included 0.5×, 1×, 2×, 3×, 4×, 5×, 6×, 8×, 10×, 12×, 15×, 18×, and 20×, providing a total of 13 different sequencing depths for each sample. By analyzing these samples at different depths, the effect of sequencing depth on subsequent analyses could be clarified.

### 4.3. SNP Calling

The BAM files, which represented samples with different depths, were imported into SAMtools for constructing a reference index, converting formats, and sorting reads. SNP calling was performed using a Genome Analysis Toolkit acceleration software named GTX.CAT (version 2.1.0). By utilizing haplotype assembly of active mutation regions, both SNPs and INDELs were simultaneously classified, resulting in the generation of VCF (Variant Call Format) files at different depths. To streamline the analysis, one-step joint typing was performed in GTX.CAT using the gtx joint command. The combined VCF files, including 31 individuals, were generated, and a total of 13 VCF files were obtained for different sequencing depths.

### 4.4. Filtering

After SNP calling, a stringent filter was used to remove possible false positive SNPs. According to the GATK (version 4.3.0.0) manual, the following filters were applied for SNPs: QD < 2.0||FS > 60.0||MQ < 40.0||HaplotypeScore > 13.0||MQRankSum < −12.5||ReadPosRankSum < −8.0. Variants used for further analysis were processed using VCFtools (version 0.1.17) [52] with—max-missing 0.95—maf 0.05—mac 3—min-alleles 2—max-alleles 2—minQ 30. This additional filtering is designed to exclude loci with low quality, non-bi-allelic data, and missing allelic information, ensuring the quality of the SNP dataset and improving the reliability of subsequent analyses.

### 4.5. Relationships

Using 20× sequencing data, a genomic relationship matrix was constructed in R (version 4.1.0) and subjected to principal component analysis (PCA). A heatmap was then generated to depict the genetic relationships between individuals, enabling the calculation of genetic correlations among them.

### 4.6. Variant Annotation

The identified SNPs were further annotated using ANNOVAR (https://annovar.openbioinformatics.org/en/latest/, accessed on 6 March 2024) [53]. The distribution of SNPs on the genome was counted and the number of SNPs in exonic regions was counted. Variants that met certain conditions were identified as potential loss-of-function (LoF) variants. These potential LoF variants were commonly labeled as “stopgain”, “stoploss”, “frameshift”, “splice”, and “nonsynonymous SNV”.

### 4.7. Comparison of Sequencing Data with Different Depths

We examined the data at various depths in terms of the number of SNPs discovered, the accuracy of genotyping, and the quality of variations assessed by transitions/transversions ratio (Ti/Tv). The SNP calling rate was represented as the ratio of SNPs, which was the number of SNPs identified in the corresponding sequencing depth divided by the number of SNPs identified in the 20× data. The SNP genotyping accuracy was represented as the proportion of consistent genotyping loci between low-depth data and the 20× data. The SNP data in VCF format were converted to PLINK (version 1.9) binary format using VCFtools (version 0.1.17), and SNP calling accuracy was determined using Python (version 3.10). Based on this, we calculated which type of detection (heterozygote/homozygote) was more likely to be unreliable in the inconsistent fraction of low-depth sequencing. The ratio of observed transitions (between purines or pyrimidines) to transversions (between purines and pyrimidines) is known as the Ti/Tv. VCFtools was also used to determine the Ti/Tv.

## 5. Conclusions

The study evaluated the impact of sequencing depth on data quality and variant detection in whole-genome resequencing of *Litopenaeus vannamei*. In order to obtain high-precision SNP information, a sequencing depth of at least 10× is required. Nevertheless, if the objective is to analyze population structure without requiring a substantial number of SNP markers, a sequencing depth of approximately 6× is a more cost-effective approach. The parameters and insights derived from this study can serve as a reference for genotyping in other species, demonstrating that high-precision SNP calling can be achieved within an adequate sequencing depth.

## Figures and Tables

**Table 1:** Summary of whole-genome resequencing data for all samples.

Population	Sample	Clean Base (G)	Effective Rate (%)	Depth (×)	Q20 (%)	Q30 (%)	GC Content (%)
GT	s3442M	54.33	99.10	33.96	90.72	86.80	44.84
GT	s3416F	51.81	99.54	32.38	91.36	88.17	47.58
GT	s3391M	62.91	99.43	39.32	91.57	88.37	45.85
GT	s3389M	55.26	99.54	34.54	91.72	88.62	44.27
GT	s3389F	58.09	99.54	36.31	91.18	87.95	44.17
GT	s3357F	56.36	99.52	35.23	91.67	88.56	44.49
GT	s3352M	63.62	99.62	39.76	90.59	86.86	45.00
GT	s3351M2	50.50	99.61	31.56	91.56	88.47	47.58
GT	s3351F2	70.57	98.84	44.11	87.75	83.67	54.67
GT	s3351F1	69.71	99.60	43.57	89.65	85.88	43.57
GT	s3332F2	63.09	99.63	39.43	89.32	85.38	44.56
SIS	s16SISM1	81.20	99.14	50.75	92.12	86.75	43.12
SIS	s16SISF1	74.93	99.07	46.83	92.43	87.17	43.01
SIS	828-2	52.65	99.94	32.91	92.73	85.00	39.59
Mex	Mex21F	53.32	99.50	33.33	91.47	87.93	43.64
Mex	Mex14M	55.06	99.09	34.41	91.42	87.84	43.73
Mex	s3M	56.85	100.00	35.53	88.41	79.34	45.95
Mex	s5M	45.77	100.00	28.61	88.95	80.14	44.41
Mex	s6F	58.67	100.00	36.67	90.46	83.36	46.81
Mex	s7F	56.93	100.00	35.58	90.70	83.81	46.55
E	EM14	86.70	99.73	54.19	91.82	85.57	42.84
E	EM13	78.42	99.16	49.01	92.60	87.40	42.42
E	EM12	84.96	99.73	53.10	91.56	85.65	41.96
E	EF11	79.82	99.39	49.89	92.41	87.11	42.61
E	EF9	79.32	99.05	49.58	92.55	87.32	42.45
E	EF8	74.40	99.04	46.50	92.60	87.39	42.45
CP	s16ZDM1	72.81	99.03	45.51	92.15	86.70	42.97
CP	s16ZDF1	57.31	99.15	35.82	88.84	75.91	43.03
CP	HYZD-2	62.80	93.34	39.22	95.86	87.39	40.20
CP	ZDWC-2F	51.93	92.34	32.46	95.54	86.55	41.41
CP	ZDWC-1F	61.05	90.43	38.16	95.62	86.76	41.28

Population: These individuals were from a total of five taxa. GT: Broodstock from Guangtai NO.1; SIS: Broodstock from Shrimp Improvement System Company; Mex: Wild population from the coast of Baja California Sur, Mexico; E: Broodstock from Ecuador; CP: Broodstock from Charoen Pokphand Foods Company. Clean Base: the amount of effective data after filtering, defined as the number of sequenced sequences after filtering multiplied by the length of sequenced sequences. Effective Rate: the ratio of clean data to raw data obtained after filtering. Depth: Sequencing depth. GC Content: the number of bases G and C combined as a percentage of the total number of bases. Q20, Q30: the percentage of bases with a Phred value greater than 20 or 30, where Phred = −10log10(e), and e is the error rate.

## Data Availability

All data have been included in the manuscript.
